# Prof. Hamilton’s New Views on Provisional Callus

**Published:** 1853-05

**Authors:** 


*Prof. Hamilton's new views on Provisional Callus.*—Prof. Hamilton's paper on the provisional callus has excited much attention. His observations, with those of Mr. Paget, will probably effect a radical change in the current doctrine relative to the union of fractured bones. So in every branch of medical science, old opinions based on authority alone are being subjected to the scrutiny of rigorous analytical investigation, which, while it confirms many truths, also discovers a multitude of errors.

We copy, with pleasure, the following extract from a review of Prof. Hamilton's memoir, by the editor of the New Hampshire Journal of Medicine:

"We cannot deny ourself the pleasure of expressing our warm admiration of the manner in which Prof. Hamilton has asserted his claim to equal originality with Prof. Paget, in proving the incorrectness of the notions usually entertained on this subject. With a manly straight-forwardness he states the facts concerning the previous publication on the part of Mr. Paget, and while he does not attempt to take a leaf from his equal's laurels, asserts on his own word, which is sufficient, that he is entitled to the same honor, and claims his reward. He accompanies his article by a large portion of Paget's lecture, that that gentleman's views may be distinctly understood. We confess we do not know which to admire most, the originality of mind displayed in making these investigations, or the gentlemanly manliness with which the apparent rival is treated."

---

*Delinquent Subscribers.*—The editor of the New Jersey Med. Reporter, in commenting on an advertisement of a "Physo-Magnetic doctor" proposing to prescribe for distant patients, after examining *a lock of their hair*, remarks that a similar testimony of the existence of some of his subscribers would afford him some gratification!

